# Qualitative Study on Dialogic Literary Gatherings as Co-creation Intervention and Its Impact on Psychological and Social Well-Being in Women During the COVID-19 Lockdown

**DOI:** 10.3389/fpubh.2021.602964

**Published:** 2021-03-18

**Authors:** Laura Ruiz-Eugenio, Ana Toledo del Cerro, Sara Gómez-Cuevas, Beatriz Villarejo-Carballido

**Affiliations:** ^1^Department of Theory and History of Education, University of Barcelona, Barcelona, Spain; ^2^Department of Sociology, University of Barcelona, Barcelona, Spain; ^3^Faculty of Psychology and Education, University of Deusto, Bilbao, Spain

**Keywords:** co-creation, dialogic literary gathering, health promotion, adult education, COVID-19

## Abstract

**Background:** Dialogic Literary Gatherings (DLG) are evidence-based interventions implemented in very diverse educational and health settings. The main elements that make DLG a co-creation intervention and promote health during the COVID-19 crisis lockdown are presented. This study focuses on the case of a DLG that is being promoted by an adult school in the city of Barcelona.

**Methods:** This qualitative study was conducted using a communicative approach. Seven in-depth interviews with participants in the online DLG have been conducted. Five of them are women without higher education ranging from 56 to 85 years old and two are educators of this school.

**Results:** The main results are 2-fold. First, the factors that make DLG a co-creation intervention, such as egalitarian dialogue and dialogical creation of knowledge in the decision-making process, are found. Second, the results show how DLG is contributing to creating a supportive environment that breaks the social isolation of confinement and improving the participants' psychological and social well-being.

**Conclusions:** The findings from this study contribute to generating knowledge about a co-creation process between adult education participants and educators in education and health promotion during the COVID-19 lockdown, which could be replicated in other contexts.

## Introduction

The COVID-19 pandemic has already caused more than half a million deaths worldwide. Although the USA, Brazil, Mexico and the United Kingdom are the countries with the most deaths, if the ratios for countries with populations >1 million are calculated, Spain would have the third highest death rate with 60 deaths per 100,000 people, behind Belgium with 86 and the United Kingdom with 69 deaths per 100,000 people, according to data updated on 9 August 2020 (1).

On 15 March 2020, a state of emergency in Spain went into effect and, as in many other countries in the world, a mandatory quarantine was established. The restrictions also included the closure of schools, universities, nonessential businesses, and any leisure establishments. On June 21, the last extension of the state of emergency in this country ended, starting what has been called the new normal.

There is still very little evidence of the impacts of the COVID-19 pandemic lockdowns on physical and mental health in older people, as well as the impacts of health and well-being interventions developed by community-based organizations to address these effects (2, 3). However, there is substantial previous evidence on social isolation and loneliness as risk factors strongly related to depression, anxiety and cognitive decline affecting the psychosocial well-being and health conditions of older adults (4–6).

For this reason, an urgent global call for action to mitigate the consequences of the lockdown on the physical and mental health of older adults has been expressed by international organizations and the scientific community (7–9). The WHO points to anxiety and feelings of isolation as factors that deteriorate people's mental and physical health since people are without the closeness of loved ones, family routines and support networks, increasing the perceived risk of death and illness (10). The UN recently launched a policy brief on the impacts of COVID-19 on older people, including measures strengthening social inclusion and solidarity during physical distancing, increasing their access to digital technologies, and their participation in decision-making processes for policies and interventions that affect their lives (11). Preliminary studies have analyzed evidence-based approaches that can address the problems of social isolation and loneliness in older people, including promoting social connections as a public health message, mobilizing the resources of family members and community networks, and developing innovative technology-based activities to improve social connections (5, 12).

Co-creation processes and participatory community-based approaches to design and implement health promotion and education interventions can contribute to effective responses at this historic moment of unprecedented global challenges created by the COVID-19 pandemic (13). Participatory approaches including end-users and stakeholders in public health research and health promotion and education have been increasingly implemented over the past two decades. There is much literature on how these participatory methods have been developed and specifically on how end-users have been included in community-based participatory research. Different concepts have been used for these approaches that also have different emphases. One concept is the co-creation of evidence, defined as the approach that integrates the best existing research evidence with other types of evidence available such as patient expectations (14). Some have deepened approaches in which existing research evidence is combined with the knowledge and experience of the directly affected communities, creating evidence from intersubjective knowledge and egalitarian dialogue (15). These approaches have promoted processes known as the Dialogic Recreation of Knowledge (DRK) in those dialogues that recreate the existing evidence on interventions that improve the living conditions of communities to respond the priorities and needs of a particular community (16) or involve end-users and stakeholders in the whole research process and the implementation of the intervention for a greater social impact (17–19). Some studies focus on how to include the most vulnerable groups, such as the elderly, in the co-creation process. Examples include the design of interventions to reduce sedentary behavior and promote physical activity (20); the design of local smoking cessation services including focus groups with smokers and ex-smokers with long-term illnesses, serious mental health problems and minority ethnic communities (21); and the promotion of community resilience processes regarding how a community addresses emergencies and other persistent and emergent threats such as severe weather and dangerous exposure to it (22).

The Dialogic Literary Gathering (DLG) is an educational intervention for the collective creation of knowledge and meaning based on the reading of the best literary creations of humanity and the subsequent dialogue between all the participants (23). Longitudinal studies carried out for more than 10 years with people close to the elderly have shown that the mortality of regular readers is reduced by 20% (24). Other studies have found that reading quality literary works increases the capacity to better understand others, facilitating empathy and pro-social behavior; in contrast, popular fiction does not stimulate this capacity (25, 26).

The first DLG was initiated in 1980 at the same adult school that has participated in this study. Currently, there is a DLG movement that involves more than 9000 pre-school, primary, secondary and adult education schools in Europe and Latin America in the framework of the Schools as Learning Communities and the extension of the Successful Educational Actions projects (27–31). As well as the DLG have been transferred to prisons and primary care health centres in Spain. There is extensive evidence on how the implementation of the dialogical learning principles on which DLGs are based promotes the cognitive, social, and emotional improvement of participants, regardless of their age, educational level and cultural background (32–37). Research that presents how online DLGs have been implemented in primary and secondary schools in Spain to promote supportive environments during the COVID-19 pandemic lockdown has been published very recently (37).

There are two goals of this preliminary study. First, the study aims to identify the elements that have been part of the co-creation decision-making process between educators, volunteers, and participants that drives the recreation and implementation of an evidence-based intervention, the DLG, during the lockdown from April to July 2020. The DLG was held to promote social connection and well-being in a group of adults and older adults of an adult school in the city of Barcelona. Second, this study seeks to collect DLG participants' perceptions of the impacts on their psychological and social well-being.

## Materials and Methods

This section first, outlines the ethics statement. Second, the hypotheses and research objectives are presented. Third, the contextualization of the Adult School La Verneda-Sant Martí and the DLG is introduced. Finally, the process of data collection and analysis using interviews with a communicative approach is explained.

### Ethics Statement

This study has been approved by the Ethics Board of the Community of Research on Excellence for All (CREA). The study has followed the official guidelines for ethical issues of the European Union H2020 research program and the Declaration of Helsinki (38) for informed consent, data protection and privacy.

Before starting each interview, the researcher informed the participants about the voluntary nature of the involvement, the aim of the study, and the confidentiality and anonymity of the information collected with the sole objective of developing the study. Participants were informed of their right to stop being part of this research at any time before the publication of the results. In the informed consent, permission was requested for the audio recording of the interviews and the publication of the study results.

### Hypothesis and Research Objectives

The hypothesis of this study is that DLG has a positive impact on psychological and social well-being in women involved during the COVID-19 lockdown.

This study responds to two research objectives:

(1) To identify the elements that have been part of the co-creation process between educators, volunteers, and participants that drives the recreation and implementation of the online DLG during the lockdown from April to July 2020.
(2) To collect the perceptions of the educators and participants on the impacts of DLGs on psychological and social well-being.

### Bibliographic Sources for Contextualization the Case

In order to contextualize the adult school in which the study was conducted, a review of the articles published in indexed journals on this school was carried out. The works of authors who form part of the theoretical basis of DLG have also been reviewed.

### The Verneda-Sant Martí Adult School and the DLGs

The Verneda-Sant Martí Adult School (39) is named after the neighborhood of Barcelona in which it is located. This school was created in 1978 through the initiative of a small group of residents in the neighborhood. With the end of the Franco dictatorship in Spain, a seven-story building that had belonged to the female section of the dictatorial movement was left empty in the neighborhood. Neighbors organized to advocate that the building be used as a space for social, health, cultural and educational projects. At the time, illiteracy rates among the population, especially women, were very high. In the end, the building hosted a nursery school, a library, different social and health services, and the Verneda-Sant Martí Adult School.

Since its inception, this adult school has been a key entity in the social and cultural transformation of the neighborhood. Together with other entities, it promoted a neighborhood movement through which joint actions have been coordinated from different spheres, such as the campaign “Let's break the silence against gender violence” in which social, educational and health organizations are involved, that is still alive now. This school has always worked in coordination with the neighborhood's primary healthcare centre. It is common for the doctors at this healthcare centre to refer cases of old people with mental health problems, such as depression, to this school. Recently, a DLG overseen by the adult school has been started in the healthcare centre. The DLG is coordinated by a retired nursing assistant who is also a participant in one of the DLGs conducted in the adult school. The impacts of participating in the DLG on the health of the people from the healthcare centre will be the subject of future studies. The present study only focuses on the online DLG developed in the scope of the adult school during the lockdown.

Two of the main characteristics that define this school since its foundation are the following: (1) All the actions that it implements are based on the best existing evidence on which actions improve the learning, well-being, values, emotions and feelings of adults and (2) It is a democratic school in which end-users participate in all decision-making processes and the needs of the most vulnerable groups such as people without basic academic qualifications are favored. Due to these reasons, this school is managed by two associations of participants, Ágora and Heura, where the latter represents women. In this school, the word “participant” is used instead of “end-user” or “student” when referring to adults who do not have higher academic degrees and participate in school activities, including all decision-making spaces. In addition to the team of educators hired by the two associations, this school has more than 100 volunteers. The volunteers include university professors who started the project 40 years ago as neighbors and educators of adults, as well as women who have become literate in this school and who now teach other people, among other very diverse profiles.

The model of democratic education for adults of the Verneda-Sant Martí Adult School is known by the scientific community in the educational field. It was the first Spanish pedagogical experience published in the Harvard Educational Review (40), and it is also the subject of other studies published in internationally prestigious academic journals such as the Teachers College Record of Columbia University (41).

One of the successful educational actions carried out at this school since 1980 is the DLG. A DLG is a space for the collective creation of knowledge through the best literary works that humanity has created. A DLG is based on dialogic learning, an evidence-based approach that collects the interdisciplinary contributions of learning sciences that focus on interactions and community involvement (23, 42–47). Its operation is based on the choices of the participants reading the literary work. The number of pages to be read before the session is agreed upon. Each person reads those pages and selects a paragraph. DLG sessions are normally held once a week with a duration of one-and-a-half to 20 h. During the DLG session, the participants stand in a circle. One person is the moderator. This person asks if there are people who want to read their paragraph, and then sets the turns in which they speak. The first person begins to read the paragraph and explains why it was chosen. Opinions do not try to explain what the author meant. The person who reads the paragraph explains what that paragraph has evoked, be it an opinion or a reflection. Anyone else who wants to comment on the paragraph read is allowed a turn to speak. When the discussion of that paragraph is over, the next person, in turn, reads his or her paragraph. This same procedure is repeated until all the paragraphs are read. One of the fundamental criteria of a DLG's operation is respect for all the opinions and different ways of thinking.

As in every school in Spain, this school was closed on 13 March 2020 due to the physical distancing measures decreed in Spain during the COVID-19 pandemic. With the closure, all face-to-face activities, including DLGs, ceased. For many of the participants, most of them older women, some of them living alone, that meant the abrupt disappearance of one of their main weekly social activities.

### Data Collection and Analysis: Interviews Using a Communicative Approach

This qualitative study has conducted seven interviews, including interviews with five women who participated in the online DLG during the lockdown and two educators who have moderated and provided technical support to the DLG, using a communicative approach. The interviews were collected from July 16th to 20th when Spain was no longer in a state of emergency and the lockdown was over. Nevertheless, it was decided that the interviews be conducted through video calls as a preventive measure to minimize the possibility of COVID-19 infection. The criterion for selecting the interviewees was their willingness to take part in the study.

It should be noted that there was already close collaboration between the research team and the school. Some of the researchers on the team have collaborated with this school for more than 10 years, and this collaboration took place for 20 years for some of them. The researchers explained the study proposal to the coordinator of the educators' team. The coordinator then informed the DLG participants of the researchers' proposal and requested their participation. All the people agreed with the study. Among these people, five participating women, in addition to the two educators, volunteered to participate in the study. The profiles of the people interviewed, and the codes assigned to them are listed in Table 1.

**Table 1:** Participants' profiles.

**Profile**	**Code**	**Gender**	**Age range**	**Academic level**
Educator	E1	Male	45–50	Higher education
Educator	E2	Female	25–30	Higher education
Participant	P1	Female	70–75	Basic education
Participant	P2	Female	70–75	Basic education
Participant	P3	Female	55–60	Basic education
Participant	P4	Female	55–60	Basic education
Participant	P5	Female	80–85	Basic education

The interviews collect the postulates of the communicative methodology of research (15, 36, 48). The interviews using a communicative approach are conducted as open conversations in which the researcher shares the existing evidence with the interviewee. In turn, the person interviewed contrasts this evidence with his or her experience. Through an egalitarian dialogue between the researcher and the researched person, an interpretation of reality is reached. In the egalitarian dialogue, the force of the arguments prevails and there is no power relationship (46). An interpretation is considered to be valid not because of the position of the power of the person who performs it, such as the researcher over the investigated person, but rather because of the strength of the arguments on which it is based, regardless of who makes that argument.

In the interviews, evidence on the elements that make up a co-creation process and the factors that contribute to improving the psychological and social well-being of people in lockdown situations was shared. Thus, a dialogue was established in which the participants contrast this evidence with their experience.

The interviews were recorded and kept in a folder on the cloud of the University of Barcelona that was shared with the members of the research team. The main contributions for each of the two research objectives were transcribed. These contributions were turned into an Excel document shared by the research team. The citations that correspond to elements that had been part of the decision-making process between educators and participants, the process of implementing the DLG and the impacts on the psychological and social well-being of the participants were classified. Once the information was analyzed, the results were sent to all the participants. In some cases, in the feedback, the participants specified some of the information given. All the participants agreed with the final interpretation of the results obtained and the conclusions.

## Results

First, the key elements of the co-creation decision-making process for the recreation and implementation of the online DLG during the lockdown are presented. Specifically, these elements include how the decision process was carried out and how the participation in the online DLG of the maximum number of people was promoted. Second, participants' perceptions of the impact that their involvement in the DLG has had on their psychological and social well-being during the confinement are collected.

### Dialogic Literary Gathering as Co-creation Intervention to Promote Psychological and Social Well-Being During the COVID-19 Lockdown

#### Co-creation in the Decision-Making Process Between Educators, Volunteers, and Participants

As in much of the world, the closing of schools was a shock to both educators and participants. For the women who participate in the weekly DLG, most of whom are older and live alone, it meant the sudden loss of one of their main social activities. Educator 2 is the coordinator of the educators' team and has been working in this school for 4 years. She explained that the democratic decision-making background of this school helped to address the situation. A few days after the closure, a video meeting was held to assess the possibilities of continuing online activities during the confinement. Educators, volunteers, and participants were involved in this encounter.

Some of the volunteers are social sciences and education researchers seeking to overcome inequalities. One of these female volunteers expressed the importance of continuing the school's activities during the lockdown. As an academic, she offered evidence of the benefits of promoting online meetings as supportive environments to break feelings of isolation. The female participants were clear from the beginning that something had to be done despite the difficulties. Educator 2 states how the team of educators alone would not have been able to do everything that was finally done. Some of them had also never used platforms for video meetings and online classes.

It was not only the proposals of the academic volunteers and the commitment of the educators that made proposals that allowed the school to overcome the situation. The participants also contributed valuable ideas so that online connections could be possible. The participants knew what specific help they needed to be able to connect to online activity. Educator 2 explained how participant 2 and others proposed very useful ideas on how the DLG could be carried out online. Some of the ideas were to make a short and very simple guide on how to download and how to use the video meeting app, to make the guide available to everyone, and to guarantee technical support for each connection through phone calls to whoever needed it. It was agreed that educators and volunteers would ensure this process as much as possible. This is how Educator 2 describes it:

We educators, of course, could not have done it alone. Some of us were also afraid of technology. There were a lot of people who finally got connected that had never participated in a video call, nor almost knew how to use their smartphone. Two important things happened: one is to have volunteer advisors, who have a lot of experience in adult education, and know the school very well. They were the ones who said at the beginning, “something has to be done here”; and two, the participants, like P2, were the ones who said, “we can do it this way”. So yes, it was super joint. What we did was to get it going and ensure technical support. Without those two things, advisors and participants, it wouldn't have been possible (E2, 04:05).

Another decision agreed upon at that meeting was that conducting the DLG would be a priority. Specifically, it was decided that two DLG sessions would be held, one session for each of the 2 weeks of confinement decreed by the government of Spain. The argument behind this decision was shared by educators, volunteers, and participants alike. This argument is based on existing evidence that a DLG is an educational action of collective knowledge creation that improves learning, values, emotions, and feelings. All agreed that the online DLG was an ideal activity to perform during the lockdown.

The next decision focused on which literary work would be read for those two DLG sessions. Some of the women participate in one of the DLGs that are traditionally held in this adult school. Therefore, they already have a list of works that they would like to read for the first time or in some cases read again. Among the different proposals they made, it was finally decided to read three stories from *The Arabian Nights* for the first session. It was considered that since the stories were short, only a few could be selected for discussion in each session so that a story would not be only partially discussed during the session when it was not yet known how long the confinement would last.

The first session of the online DLG was held on Saturday, April 11. It was so successful that the participants asked that it be held weekly during the weeks of confinement, and it was. At that time, it was already known that the state of emergency would be extended. Finally, a DLG was held every Saturday from 6 to 7:30 p.m. until June 20. During that period, all the tales of *The Arabian Nights* were finished. The next day, the state of emergency was lifted. However, the new normality did not allow the return to normal activity in the adult school either.

Even though the academic year in Spain ends that week in June, this adult school, since it is managed by the associations of participants, decided years ago to be open the month of July as well. Under the slogan “The school opens in July,” a range of cultural and training activities are held every July. In a second meeting, it was decided to continue with the DLG online for the whole month of July. This time they chose to read the *Chronicle of a Death Foretold* by Gabriel García Márquez. This is how educator E1 explains it:

A proposal of different classical works was made by the participants when *The Arabian Nights* were finished. I was in the process of choosing the book to read in July. Different works were proposed, and now we are reading from García Márquez: *Chronicle of a Death Foretold*. Both the participants and we made proposals; the proposal that came out was from a participant who said that it was a book that could be easily finished in July (E1, 11:50).

#### Promoting the Participation of as Many People as Possible in the Online DLG

Once the decision was made to carry out the online DLG, the priority was that the activity reached as many people as possible. For this purpose, a phone call was made to all the people participating in the different DLGs of the school. This was done to reach all the people since many of them do not have e-mail or online social network accounts or do not use them assiduously. Educator 1 (3:00) explained how these calls would be the educators' first contact with many of the participants since the confinement began. There was a concern for the participants' well-being and knowing how they were doing. Many of them needed to talk and share how they were feeling and how they were living during the confinement situation. They were then informed that the DLG would be conducted online and how they could connect to the Zoom video meeting app where it would take place. They were also given simple guidelines on how to use Zoom on their computer or mobile phone. This educator explained how they were sensitive to the issues related to these calls so that no one would feel guilty about not being able to participate, especially at a time when some people were very focused on how the pandemic was developing and some even already had a relative who had COVID-19.

Educators and volunteers were available through WhatsApp or by phone to provide technical support to those who needed it to connect to Zoom. During the development of the DLG online, one of the educators moderated the discussion and other people, sometimes educators and volunteers, were available in case someone during the connection needed technical support and a call had to be made. Educator 1 explains how they logged in before the start time of the DLG to provide this support. He stated that the technical support was hardly needed when the different sessions of the DLG were taking place.

On DLG day, we used to go online earlier to provide technical assistance. During the discussion, I was the moderator, but there were other people, one or two, who acted as technical support. In the end, people were doing very well, but if there was a problem a call was made (E1, 18:30).

Educator 2 explained (E2, 08:50) that another of the decisions made to promote participation was that there should be a free online version of the book chosen. If this had not been the case, it would have been difficult to participate because the libraries were closed or people would have to buy a book under the circumstances of the confinement, which would have been an additional difficulty for older people who do not usually buy items online, in addition to the economic costs. Finally, ~30 people participated in each online DLG session. In each of the sessions, the dialogical criteria with which the traditional DLG worked were maintained. This implied that the moderator at the beginning of each session reminded the participants of the importance of respecting different opinions, keeping their turn to speak and that priority will be given to people who had not yet spoken to facilitate the participation of those who have more difficulties to intervene. This is how educator 2 explains it:

The interaction has been the same (as in the traditional DLG) because what was done when moderating was the same. Well, first, we all learned that we had to have the microphones off and only the microphone of the person who had the floor was open. After having made it clear that and how to request the floor, the operation has been the same as in the traditional DLG. The moderator explained the criteria and asked who had a paragraph to read. Then, people raised their hand, the moderator noted. The moderator gave the floor to the first person. The first person reads his or her paragraph and explains why he or she had chosen it. Then, the moderator opened the floor again in case anyone wanted to comment on the paragraph read. This continued until all the paragraphs were finished (E2, 11:30).

### Impact of the DLG on the Psychological and Social Well-Being of Participants During the COVID-19 Lockdown

Both the educators and the female participants in the interviews stated that participating in the DLG during confinement has had a positive impact on the participants' psychological and social well-being; some participants have even reported that they have felt better physically. The factors in the online DLG that participants perceived as having a positive impact on their well-being are presented.

#### Good Literature, Egalitarian Dialogue and Respect for the Others' Opinions Improve Psychological and Social Well-Being

Respect for others' opinions, even if the opinions are different from one's own opinions, is one of the criteria for the functioning of the DLG and one of the factors that all the participants pointed out as having a positive impact on their well-being. There is previous evidence that the reading of quality literary works such as universal classics increases individuals' capacity to understand other people, facilitating empathy and pro-social behavior, by deepening the psychology of the characters and the reasons for their actions. In the DLG, this evidence can be corroborated, and it is also reinforced by the dialogues that are given on fragments of the text. P1 asserts that having participated in the DLG has improved her mental and physical health before and even more during the lockdown. She states that a feeling of freedom is created by the atmosphere of respect for all opinions that counteracts the limitations of the physical distancing. This is what she said:

The gatherings have improved my mental and physical health during the lockdown and before. At the gathering, reading the book is different. An atmosphere of freedom is created because while you respect what others say, they also respect you. It has always helped me and in the lockdown more because you are very limited without being able to get out and meet other people (P1, 13:00).

P3 also refers to the fact that the DLG pushes her to socialize and better manage her human relationships, thus feeling more encouraged. This is how she explained it:

It makes you feel more encouraged. Always the psychological, or the emotional, is related to the physical, it makes you have another drive. It pushes you to face human relationships (P3, 09:58).

P4 reflected on how literary quality books, such as the one read in the DLG, make her think and have “touched her inside.” Her affirmation is in line with existing evidence on how the simple reading of a good book changes the brain by increasing neural connections (49–51). She adds that it makes her feel alive and see life from a new perspective:

All good books touch you inside. They deal with issues in a deep way that makes you think, see life differently. People who don't have a taste for reading don't know what they're missing; for me, reading brings a lot of life to me (P4, 19:30).

#### DLG as a Supportive Environment Overcoming Feelings of Isolation and Improving Self-Esteem

Educator 1, who moderated all the sessions of the online DLG, explained some of the assessments that the participants have made. They said that the online DLG was “like a window open to the world” (E1:14:10) or “like meetings in which we became more and more friends every week” (E1, 16:00). E1 claims that the DLG during the confinement has allowed the participants to continue to have contact and created an environment in which they felt even more united than before (E1: 14:40). The creation of supportive environments and the promotion of a sense of community during confinement have been identified by the recent scientific literature as two of the elements that have contributed to lessening the feeling of isolation (2). Many of the participating women live alone. The DLG has been one of the activities that have helped them overcome the feeling of isolation and minimize the distress of the situational uncertainty, fear of illness, and stress caused by listening to the daily news or because of the amount of misinformation that has spread through social networks (52). This is how P2 who lives alone explains it:

It helps you mentally because you can talk to someone. It's not the same to see people on the screen and be in dialogue with them than to receive a message from WhatsApp. Because there is stress... you think when you wake up: let's see if the news has improved and it doesn't, on the contrary, every day it was worse. DLG has really helped me. If the world had totally shut down, if everything had gone silent, it would have been harder. At least, the hour or 2 h of the DLG helped me (P2, 21:00).

Some of the participants and educator E2 affirmed that the supportive environment that has been created in the DLG has helped to increase self-esteem. The fact that the educators demonstrated confidence and believed that they would be able to participate in a DLG online when some of them had very few digital skills helped them to believe in themselves and to think that they were capable of doing it, thus, increasing their self-esteem when they saw that they had finally achieved it. Educator 2 (14:40) and (participant 1 03:20) commented that in the beginning, it was challenging for the latter to connect, but as the weeks passed and she had the support of the educators, volunteers and other participants of the DLG, these difficulties disappeared. Participant 5 also explains how she has received support from educators, volunteers, and other participants in the DLG online. She claims that this support has had an impact on improving her self-esteem and well-being:

The school and the DLG is one of the places that have helped me the most. It's like raising my self-esteem, because when you think you can't, and you don't believe in yourself, but suddenly there's someone who believes in you, you grow up and you try to better yourself and in the end, you succeed (P5, 17:40).

## Discussion and Conclusion

This preliminary study is the first to analyse the impact of a co-creation intervention, an online DLG, on the psychological and social well-being of women and older women during the lockdown period of the COVID-19 crisis. Specifically, seven interviews using a communicative approach were carried out with women and educators who participated in this intervention within the framework of the activities organized by an adult school in a neighborhood of Barcelona, Spain. Despite being a preliminary study that includes a few interviews, it makes two valuable contributions. First, it provides knowledge about the successful elements for co-creation within the framework of a decision-making process for the implementation of an intervention that breaks the feeling of isolation during the lockdown, especially among older women with basic levels of education and few digital skills. Second, it offers qualitative evidence on how the online DLG has had a positive impact on their psychological and social well-being during that period from participants' perspective.

This study has responded to the call to action made by international organizations such as the UN and the WHO (10, 11), as well as by the scientific community (4, 7, 53, 54), to develop public health policies and community networks to address the consequences of social isolation and loneliness on older adults during the physical distancing measures implemented due to the crisis of the COVID-19 pandemic. Social isolation and loneliness are important risk factors that have been linked to poor psychological and physical health (5). This study responds to the call by offering evidence that this co-creation intervention could be recreated in other contexts and adds to previous evidence on its potential for scalability and transferability (32, 33, 37, 55–59).

Dealing quickly and effectively with the psychosocial consequences of this lockdown on older people has made the co-creation processes of community networks implementing health promotion interventions even more meaningful in recent months. Very recently, a co-creation methodology was developed to identify those health professionals who require emergency mental health because of the COVID-19 crisis in Scotland through expert advisory groups of stakeholders (60). However, at the time of the writing of the current article, no studies had been published on the development of co-creation processes to alleviate the psychological and social consequences of the lockdown due to the COVID-19 pandemic on adults and older adults.

The debate on the impact of community-based participatory approaches in decision-making, design and implementation has intensified over the past 10 years in public health research (17, 22, 61–63). Previous literature has highlighted the difficulties of making these processes co-creative with those vulnerable groups that are the hardest to reach (20, 21). This study provides knowledge that contributes to the identification of the successful elements for co-creation in decision-making processes with vulnerable groups, such as older women with a basic level of education.

The decision-making process between the educators, volunteers and participants of this school regarding what type of activity would be promoted during the lockdown responds to what is known in public health and health promotion research as evidence-informed decision making (EIDM) (14). Some studies have pointed out that to be able to develop EIDM processes from co-creation or participatory community methodologies, relationships of trust and mutual respect between academics, stakeholders and end-users are necessary (18, 19). In the school involved in this study, these relationships exist and are maintained over time. For example, some of the volunteers who have participated in the decision-making process are academics in the fields of social sciences, education, and health promotion. Some of these volunteers have been involved with this school for years. These people are involved due to their social commitment as academics, without receiving any economic benefit, to ensure that the school maintains the principles with which it was created: the implementation of evidence-based interventions and that the decision-making processes are democratic, based on an equal dialogue between all the people involved, and always prioritize the needs of the most vulnerable groups.

The decision-making process for the implementation of the online DLG during the lockdown is based on existing evidence that this intervention is a successful educational action that improves cognitive, social and emotional well-being (33, 35, 36, 64). However, this process of decision-making and implementation goes beyond the EIDM and responds to the so-called dialogic recreation of knowledge (DRK) (16, 32, 37). In the DRK, academics together with the stakeholders and end-users of a community start with the existing evidence on educational, social and health promotion actions that have been shown to improve the living conditions of communities in very different contexts. What differentiates the DRK from other EIDM processes is that an equal dialogue is established between academics, stakeholders and end-users based on how that evidence can be recreated to address the priorities and needs of a particular community. In the case of the present study, the DLG, an evidence-based intervention, is recreated to overcome the barriers of implementing it online during the lockdown period with adults who have basic levels of education and few digital skills. A recently published study has also focused on the DRK of online DLGs and other online dialogic interventions promoted by primary and secondary schools to create supportive environments during the lockdown in Spain (37).

This DRK process is based on the existing evidence, which is provided by the academics involved in it. The contributions made by the academics, educators and participants are valid according to the arguments on which they are based and not according to power relations, such as that of a female academic regarding a woman with a basic level of education (65, 66). All those involved in that decision-making process agreed that the DLG was an ideal activity to carry out during the lockdown to break the social isolation. The arguments provided by the different parties helped to overcome the barriers to implementing such an online activity during this period. Furthermore, the contributions of the women participants were especially valuable. They knew what concrete help they needed and how they must be helped to be able to connect to a virtual platform and follow the online DLG. The predisposition of the team of educators and volunteers who collect the contributions of these women provides technical and human support that makes it possible for women with basic levels of education and few digital skills to finally be autonomous on these virtual platforms and follow the online DLG weekly.

The perceptions of the positive impact on their psychological and social well-being expressed by the participating women are in line with existing evidence already referenced on the impact of the DLG. In addition, the perceptions of some of the female participants have also pointed out that the positive impact of the DLG in breaking down feelings of isolation and minimizing the anxiety generated by uncertainty and the fear of getting sick has been even more important during the lockdown period. Thus, the online DLG contributes to minimizing the risk factors for the deterioration of the psychological and social well-being and health of older people during confinement that have already been reported in other studies (5, 7, 54).

From the participants' accounts, it has been identified that the online DLG has had a positive impact on their well-being during confinement not only because it is a virtual space for social relations but also because of the principles on which this intervention was based. The reading of quality literature, the respect for others' opinions and the respect for turns to speak generate a dialogical environment in which the participants feel free to express their opinions and reflections. They also report that the type of book they read and the sharing of the reflections that these readings evoke impact them positively by offering them new perspectives on life. These dialogues and interactions in the online DLG facilitate the creation of a sense of community and a supportive environment also identified by previous studies as key elements to overcome the feeling of isolation (67). For example, not just the educators and volunteers have provided technical support to the participants. The female participants in the online DLG have been helping each other to overcome difficulties with connections.

## Practical Recommendations

The online DLG could be a kind of co-creation intervention that can be promoted in an intersectoral partnership between clinical and community-based organizations to reduce the social isolation of the elderly and promote a supportive environment through remote connectivity, as recently suggested by other preliminary studies (2). An online DLG could be a complementary intervention to other services to engage and support older adults during the difficult periods of physical distancing from the pandemic by building on existing or creating new practices in the community. Two limitations must be considered so that this intervention can be implemented in other contexts. The first is the poor digital abilities of some older people. The second is that not all people have access to electronic devices with internet access. Regarding the first limitation, this article provides evidence on how to overcome it. Regarding the second limitation, more research is needed on the social impacts of future projects that this school is working on to obtain public and private funding to increase access to these devices among isolated older people.

## Data Availability Statement

The raw data supporting the conclusions of this article will be made available by the authors, without undue reservation.

## Ethics Statement

The studies involving human participants were reviewed and approved by Ethics Board of the Community of Research on Excellence for All (CREA). The patients/participants provided their written informed consent to participate in this study.

## Author Contributions

LR-E: contributed to the conceptualization of the study under the research line of successful educational actions overcoming inequalities that women with no academic qualifications face, in the framework of the Ramon y Cajal grant and revised and edit the final version. AT and SG-C: collected the data. LR-E, AT, SG-C, and BV-C: contributed to the formal analyses and discussion of the data. LR-E and BV-C: drafted the manuscript. All authors have made substantial contributions and have read and agreed to the published version of the manuscript.

## Conflict of Interest

The authors declare that the research was conducted in the absence of any commercial or financial relationships that could be construed as a potential conflict of interest.
